# Prenatal Diagnosis of Isovaleric Acidemia From Amniotic Fluid Using Genetic and Biochemical Approaches

**DOI:** 10.3389/fgene.2022.898860

**Published:** 2022-06-30

**Authors:** Si Ding, Lili Liang, Wenjuan Qiu, Huiwen Zhang, Bing Xiao, Liping Dong, Wenjun Ji, Feng Xu, Zhuwen Gong, Xuefan Gu, Lei Wang, Lianshu Han

**Affiliations:** ^1^ Department of Pediatric Endocrinology and Genetic Metabolism, Xinhua Hospital, Shanghai Institute of Pediatric Research, Shanghai Jiao Tong University School of Medicine, Shanghai, China; ^2^ Neonatal Disease Screening Center, Zibo Maternal and Child Health Hospital, Zibo, China; ^3^ Center for Prenatal Diagnosis, Xinhua Hospital, Shanghai Jiao Tong University School of Medicine, Shanghai, China

**Keywords:** genetic analysis, isovaleric acidemia, mass spectrometry, metabolite analysis, prenatal diagnosis

## Abstract

**Background:** Isovaleric acidemia (IVA) is an inborn error of leucine metabolism and different approaches have been applied to its prenatal diagnosis. However, systemic application of a biochemical strategy is rare. To evaluate its reliability and validity, we conducted a retrospective study of our experience with metabolite measurement together with genetic analysis in IVA prenatal diagnosis at a single center.

**Methods:** A total of eight pregnancies whose probands were diagnosed as IVA were referred to our center for prenatal diagnosis. Prenatal data of genetic analysis and metabolite measurement using tandem mass spectrometry (MS/MS) and gas chromatography/mass spectrometry (GC/MS) in amniotic fluid (AF) samples were retrospectively reviewed.

**Results:** Genetic and biochemical results were both available in these eight at-risk fetuses. Among them, two fetuses had higher levels of isovalerylcarnitine (C5) and C5/acetylcarnitine (C2) in AF compared with normal reference range and, thus, were determined to be affected, both of whom were found to carry compound heterogeneous mutations according to genetic analysis. The remaining six fetuses were determined to be unaffected based on a normal AF metabolite profile, except one showed slightly elevated C5 and they were found to be carriers according to genetic analysis. However, the level of isovalerylglycine (IVG) could not be detected at all in both groups.

**Conclusion:** The biochemical analysis, as a quick and convenient method, could be an additional reliable option for the prenatal diagnosis of IVA, especially in families with inconclusive genetic results, and can achieve a more precise diagnosis in conjunction with mutation analysis.

## Introduction

Isovaleric Acidemia (IVA, OMIM#243500) is the first organic acidemia recognized in humans. The incidence of IVA ranges hugely from 1:33282 to 1:622489 in the worldwide (Chace et al., 2003; Kasper et al., 2010; Golbahar et al., 2013), and varies from regions to regions in China, which was reported to be 1:365,000 in Taiwan (Lin et al., 2007), 1:123,400 in Zhejiang province (Hu et al., 2020) and 1:84,469 in Quanzhou district (Lin et al., 2020). IVA is an autosomal recessive inborn error of leucine metabolism caused by a deficiency of the isovaleryl-CoA dehydrogenase (IVD; E.C.1.3.99.10) enzyme (Tanaka et al., 1966). Its deficiency results from mutations in the *IVD* gene, leading to the accumulation of derivatives of isovaleryl-CoA that are detected as isovalerylcarnitine (C5), isovaleric acid, 3-hydroxyisovaleric acid, and isovalerylglycine (IVG) in the cells, blood and urine (Vockley and Ensenauer, 2006; Ibarra-González et al., 2020). Based on the clinical presentation, IVA can be classified into three distinct phenotypes: an acute neonatal form, a chronic intermittent form with late onset in childhood and a mild, potentially asymptomatic phenotype (Ensenauer et al., 2004). Patients with an acute neonatal presentation are at risk of severe, potentially life-threatening acidotic decompensations, showing a high mortality of 33%. Patients with late onset is associated with developmental delay, who can also present acidotic episodes during catabolic factors such as infection and stress and appeared to have poor neurological outcomes (Grünert et al., 2012; Kölker et al., 2015). In addition, even with early diagnosis and effective treatment, prognosis of IVA remains relatively disappointing. It has been reported that patients who have been metabolically stable for more than 1 decade were still at risk of metabolic decompensation in episodes of catabolic stress (Kimmoun et al., 2008; Szymańska et al., 2020).

Prenatal diagnosis is thus essential for the family with IVA proband to prevent the recurrence of IVA. Different approaches have been reported for the prenatal diagnosis of IVA, including genetic variant analysis of *IVD* genes in amniocytes (Tanacan et al., 2019), IVD enzyme activity assay in amniotic or chorionic villi sampling (Kleijer et al., 1995), and quantification of the characteristic metabolites such as acylcarnitines and organic acids in amniotic fluid (AF) (Hine et al., 1986; Shigematsu et al., 1991). However, each approach has its limitations. Mutation analysis depends entirely on complete genetic information from the proband and parents, while some probands were found to carry only one causative mutation, leading to the inability to make a precise diagnosis by genetic testing alone. Enzymatic analysis is available for prenatal diagnosis with cell cultivation required, which is troublesome and time-consuming. In addition, there is a risk of maternal cell contamination in above two methods which may potentially lead to misdiagnosis (Roberts et al., 1988; Hasegawa et al., 2005). With the advancing use of mass spectrometry in metabolite analysis, this biochemical approach beneficially provides a fast and convenient method for the prenatal diagnosis of many inherited metabolic disorders (Ji et al., 2019; Dai et al., 2020; Xiao et al., 2020), yet limited research is available in IVA. In this study, we describe our experience in the prenatal diagnosis of IVA. Overall, we have investigated eight pregnancies in seven unrelated families by metabolite analysis together with genetic analysis in AF.

## Materials and Methods

### Families and Probands

In this study, seven families (eight pregnancies) in which the probands diagnosed with IVA were referred to our center for prenatal diagnosis. The probands were diagnosed based on clinical symptoms, biochemical results and genetic testing of *IVD* genes. Informed consent forms were signed by the parents or legal guardians of the study participants. This study was approved by the Ethics Committee of Xinhua Hospital Affiliated to Shanghai Jiao Tong University School of Medicine (approval number XHEC-D-2021-172).

### Amniocyte Samples

In each case, 20 ml sample of AF was collected at 16–20 weeks of gestation from the pregnant women. The cells in 10 ml AF were used for DNA extraction, and supernatant samples was used for metabolite analysis by mass spectrometry. The remaining 10 ml of AF was cultured in a flask for karyotyping analysis, with the cultured amniocytes also available as a back-up. Furthermore, 3–4 ml of peripheral blood was collected from all IVA pedigree members to perform a linkage analysis and to exclude maternal cell contamination.

### Metabolite Analysis

The levels of C5 and C2 were quantitatively analyzed by MS/MS (Applied Biosystems, API 2000) using 3 μl of AF supernatant and the levels automatically calculated based on the assigned values for internal standards using Chemo View v1.2 software (Han et al., 2007). (Parameters for MS/MS analysis are listed in Supplementary Table S1) The organic acid fraction was extracted, methylated, and analyzed by chemical ionization GC/MS (QP 2010, Shimadzu Limited, Kyoto, Japan) operated in selected ion monitoring mode. For each AF supernatant sample, 2 ml of sample was mixed with stable isotope-labeled compounds and internal standards as described by Hasegawa et al. (2005). Concentrations of IVG in the AF were calculated using GC-MS Solution v2.40 software. (Parameters for GC/MS analysis are listed in Supplementary Table S2.) Standard scatter plots were generated for level of C5, C5/C2 and IVG in affected and unaffected groups using Prism 8 (Graph-Pad Software Inc.).

### Gene Variant and Linkage Analysis

Genomic DNA from the AF was extracted with a QIAamp DNA Blood Mini Kit (Qiagen Inc., Valencia, CA). Five closely linked flanking short tandem repeat (STR) markers at the *IVD* gene locus were selected to perform the linkage analysis and to exclude maternal cell contamination. We then conducted polymerase chain reaction (PCR) amplification and direct Sanger sequencing, which was performed as described in a previous paper (Lee et al., 2007). Nucleotide variations were identified using a reference sequence from Genbank (*IVD*: NM_002225).

In our study, fetuses harbored homozygous variants or compound heterozygous variants of *IVD* were diagnosed as IVA. We then used the ClinVar database, the HGMD database and the former literatures to identify whether the mutations had been reported.

## Results

The genetic and biochemical results of the eight fetal samples from seven families are listed in Table 1. Among the eight fetuses, two fetuses were judged as “affected” and six fetuses were judged as “unaffected” based on the combination of these analyses. The STR analysis indicated that no samples had maternal cell contamination. Postnatal follow-up of all these six unaffected fetuses showed a normal phenotype. For the two affected fetuses, the parents chose to terminate pregnancies.

**TABLE 1:** The prenatal results of biochemical and genetic tests analysis in the amniotic fluid samples of eight fetuses.

Fetal sample number	Metabolites of aniotic fluid			Variants in the proband (NM_002225)		Fetus status	Variant of fetus	
	MS/MS		GS/MS	Allele 1 (paternal)	Allele 2 (maternal)					
	C5 (μmol/L)	C5/C2	IVG (mmol/mol cr)							
F1^a^	0.28	0.02	ND	c.149G > A	c.1184G > A	Carrier	c.1184G > A	
F2^a^	0.24	0.02	ND	c.149G > A	c.1184G > A	Carrier	c.1184G > A	
F3	0.31	0.03	ND	c.205G > A	c.296-3_296-2delinsGG	Carrier	c.296-3_296-2delinsGG	
F4	0.946	0.08	ND	c.415C > T	c.214G > T	Carrier	c.415C > T	
F5	0.66	0.06	ND	c.359G > A	c.476G > C	Carrier	c.359G > A	
F6	**2.7**	0.072	ND	c.476G > C	c.358C > T	Carrier	c.358C > T	
F7	**3.44**	**0.237**	ND	Exon12del	c.467G > C	Affected	Exon12del	c.467G > C
F8	**4.22**	**0.48**	ND	c.548C > T	c.757A > G	Affected	c.548C > T	c.757A > G
References range	0.04–1.00	0.01–0.08	ND					

a F1 and F2 were two pregnancies from one family; elevated metabolites are shown in bold.

ND, not determined; C5, isovalerylcarnitine, C5/C2 C5/acetylcarnitine (C2), Isovalerylglycine (IVG), MS/MS, tandem mass spectrometry, GS/MS, Gas chromatography/mass spectrometry.

### Biochemical Analysis of AF Metabolites

**FIGURE 1:**
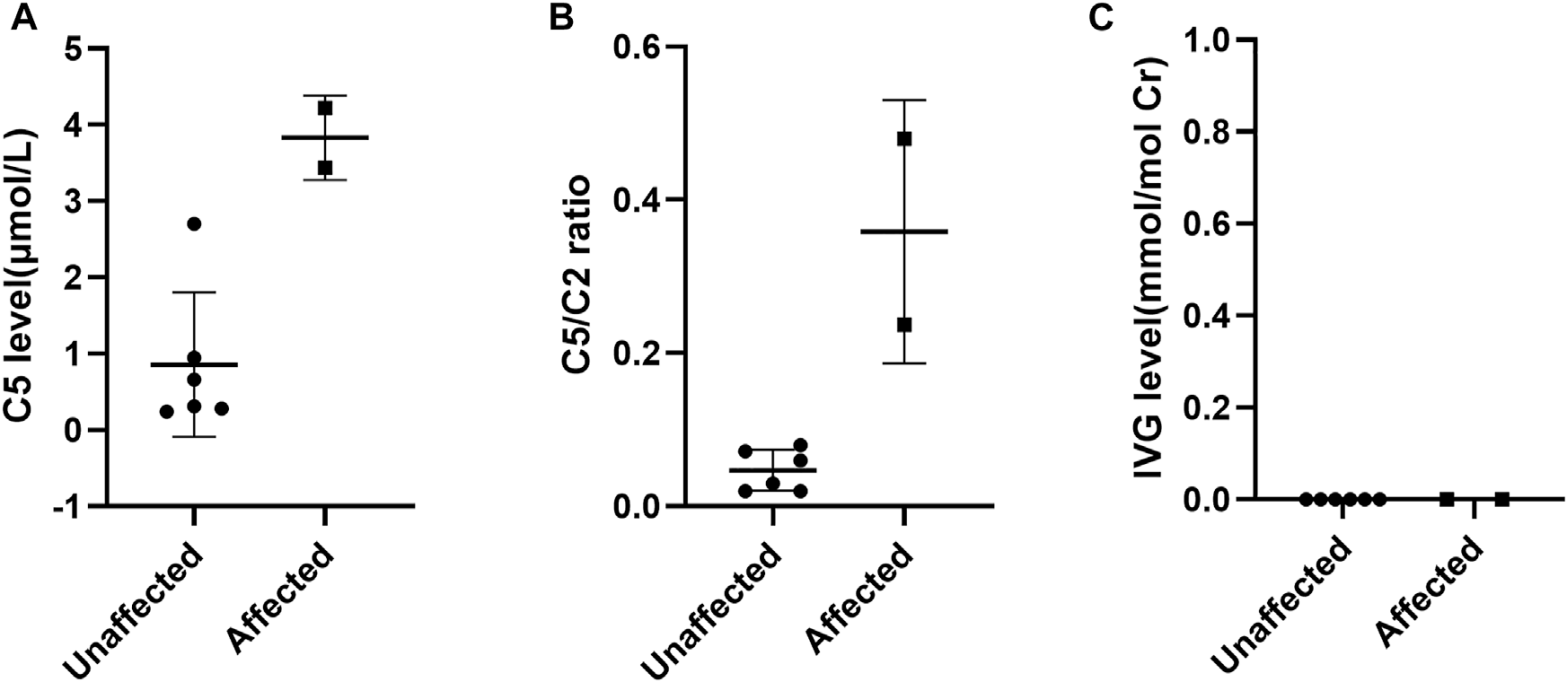
Scatter-plots of characteristic metabolite levels in affected and unaffected AFs. **(A)** The distribution of C5 in AFs between affected and unaffected samples; **(B)** the distribution of C5/C2 ratios in AFs between affected and unaffected samples; **(C)** the distribution of IVG in AFs between affected and unaffected samples.

In our study, the reference ranges of C5, C5/C2 and IVG were 0.04–1.00 μmol/L, 0.01–0.08 and 0.00 mmol/mol Cr, respectively. Fetuses with the above metabolites’ levels higher than the upper limit of reference ranges were suggested as IVA. As a result, a total of two fetuses were found to have higher levels of C5 and C5/C2 compared to that of defined reference ranges and they were thus determined to be affected. Among the remaining six fetuses, the C5 level in F6 were slightly above the upper limit of the reference range, while the level of C5/C2 were within the reference range. The other five samples showed normal levels of C5 and C5/C2. And these six fetuses were determined to be unaffected according to our metabolite test results. However, the IVG levels in all eight fetal samples could not be detected at all (Figure 1).

### Genetic Analysis of the Pathogenic Variants in Amniocyte DNA

Luckily, all the eight at-risk fetuses had clear information concerning pathogenic variants in the probands and parents. The molecular spectrum of this cohort was heterogeneous, with thirteen different variants identified: c.149G>A, c.205G>A, c.214G>T, c.358C>T, c.359G>A, c.415C>T, c.467G>C, c.476G>C, c.548C>T, c.757A>G, c.1184G>A, c.296-3_296-2delinsGG and Exon12del, most of which were missense variant and the c.214G>T, c.358C>T, c.415C>T and c.296-3_296-2delinsGG variants were novel.

In this study, a total of two fetuses harboring compound heterozygous variants, were determined to be affected. And the remaining six fetuses with a pathogenic variant were determined to be unaffected as heterozygous carriers.

### Comparison of Metabolite Results and Genetic Results

Since the level of IVG could not be detected in all samples, we mainly compared metabolite results of C5 and C5/C2 with genetic results, which showed high consistency in our study. A total of two fetuses were determined to be affected according their genetic results, both of whom carried compound heterozygous variants. These two fetuses were found to have elevated levels of C5 and C5/C2. Thus, they were also judged as affected by metabolite results (Table 1). The remaining six fetuses were determined to be unaffected by mutation analysis, all of whom were *IVD* pathogenic single variant carriers. Among them, five fetuses were found to have normal levels of C5 and C5/C2 while the other one fetus (F6) showed slightly inconsistent genetic and metabolite results (Table 1). The fetus with a pathogenic variant inherited from the mother was found to have an elevated level of C5, but a normal level of C5/C2. This fetus was determined to be unaffected based on the combination of genetic and metabolite results. Postnatal follow-up of the fetus showed a normal phenotype.

## Discussion

IVA is known as a relatively rare, potentially life-threatening inborn error of leucine metabolism. Even with rapid diagnosis and effective treatment, patients with IVA are still at risk of metabolic decompensation, no matter what the form is. The disappointing outcome will increase the families economic and spiritual burden. Thus, prenatal diagnosis is an essential strategy for the family with IVA probands to prevent the recurrence of IVA. In this study, we shared our experiences with prenatal diagnosis of IVA in eight pregnancies.

The measurement of metabolite levels in AF by mass spectrometry has been increasingly used for the prenatal diagnosis of numerous inherited metabolic disorders, yet few reported in IVA. In a notable example, Shigematsu et al. reported that metabolite analysis of acylcarnitines by MS/MS and organic acids by GC/MS in AF allowed a rapid and reliable diagnosis, while it was based on a single case (Shigematsu et al., 1991). In addition, it has been reported that IVG possibly present below the lowest levels, which remains a risk of false-negative results (Kumps et al., 2004). Therefore, systematic application and evaluation of these biochemical methods are critical. Given that genetic analysis was generally recognized as the golden standard for prenatal diagnosis of IVA, in this study, we retrospectively reviewed prenatal diagnostic data from eight at-risk pregnancies and first analyzed the reliability of the biochemical approach for prenatal diagnosis of IVA by comparing the biochemical results with the molecular results.

Among these eight cases, the levels of C5 and C5/C2 were consistent with genetic results in both affected fetuses and in five of six unaffected fetuses, these two biochemical markers were also completely consistent with genetic results. Only the level of C5 in F6 was found to show a slight discrepancy with genetic results. One of the probable reasons might be associated with the selection of the cutoff value. It has been suggested that a reference range based exclusively on normal population might lead to many false positive results. Therefore, the reference range of prenatal metabolite levels need to be adjusted in response to the overlap between normal population and disorder range (McHugh et al., 2011). However, it is difficult to establish more reasonable ranges on the basis of the small sample size in our study. The IVG assay was also performed for the prenatal diagnosis of IVA, which yet was found not to be valuable, since the AFs from all eight at-risk pregnancies contained no measurable IVG, whether they carried an affected or unaffected fetus. This is inconsistent with that described in the previous reports where the IVG levels in AFs of affected fetuses were notably 30-60 times higher than those in AFs of unaffected fetuses (Hine et al., 1986; Kleijer et al., 1995). The false negative results might be due to the low presentation of this metabolite in AFs and adsorption losses. Due to the limitation of sample size in this study, further data are needed to determine the reliability of these three biochemical markers in the prenatal diagnosis of IVA.

Mutation analysis is generally recognized as the most reliable strategy for prenatal diagnosis of IVA, which yet may potentially lead to false negative results due to maternal cell contamination. Another limitation of this method is that it depends on the availability of known familial mutations. Luckily, all the probands in our center had found two causative variants and no samples had maternal cell contamination. However, in some IVA families, not more than one causative mutation was found in the proband, or genetic testing was not performed, especially in some remote areas, this can hinder a precise diagnosis by genetic analysis alone. In this situation, metabolite analysis by mass spectrometry could be optional which is able to provide reliable results using only a small amount of sample but we must also admit that amniotic fluid testing is invasive. Additionally, the biochemical results can be achieved within 3 days, which is much earlier than that of genetic analysis taking at least 2 weeks. This enables IVA families to make a timely decision concerning the pregnancies. This advantage was observed in our previous reports on prenatal metabolite analysis in methylmalonic acidemia, propionic acidemia and glutaric acidemia-Ⅰ (Ji et al., 2019; Dai et al., 2020; Xiao et al., 2020). Therefore, biochemical analysis would offer supplementary evidence in the prenatal diagnosis of IVA families, especially when genetic results were inconclusive, providing increased diagnostic accuracy. However, the reliability of biochemical approach in the prenatal diagnosis of IVA is warranted to be further verified by expanding the sample size.

## Conclusion

In summary, prenatal diagnosis is necessary in families of probands with IVA. Biochemical analysis using only a small amount of amniotic fluid, offers fast and reliable results, which could be a suitable option in the prenatal diagnosis of IVA, especially in families without conclusive genetic results, and can increase the accuracy together with mutation analysis.

## Data Availability

The original contributions presented in the study are included in the article/Supplementary Material, further inquiries can be directed to the corresponding authors.

## Abbreviations

IVA, isovaleric acidemia; IVD, isovaleryl-CoA dehydrogenase; MS/MS, tandem mass spectrometry; GC/MS, chromatography/mass spectrometry; C5, isovalerylcarnitine; C2, acetylcarnitine; C5/C2, isovalerylcarnitine to acetylcarnitine ratio; IVG, isovalerylglycine; AF, amniotic fluid.

## References

[B1] Chace D. H. Kalas T. A. Naylor E. W. (2003). Use of Tandem Mass Spectrometry for Multianalyte Screening of Dried Blood Specimens from Newborns. Clin. Chem. 49, 1797–1817. 10.1373/clinchem.2003.022178 14578311

[B2] Dai M. Xiao B. Zhang H. Ye J. Qiu W. Zhu H. (2020). Biochemical and Genetic Approaches to the Prenatal Diagnosis of Propionic Acidemia in 78 Pregnancies. Orphanet J. Rare Dis. 15, 276. 10.1186/s13023-020-01539-w 33028371 PMC7539428

[B3] Ensenauer R. Vockley J. Willard J.-M. Huey J. C. Sass J. O. Edland S. D. (2004). A Common Mutation Is Associated with a Mild, Potentially Asymptomatic Phenotype in Patients with Isovaleric Acidemia Diagnosed by Newborn Screening. Am. J. Hum. Genet. 75, 1136–1142. 10.1086/426318 15486829 PMC1182150

[B4] Golbahar J. Al-Jishi E. A. Altayab D. D. Carreon E. Bakhiet M. Alkhayyat H. (2013). Selective Newborn Screening of Inborn Errors of Amino Acids, Organic Acids and Fatty Acids Metabolism in the Kingdom of Bahrain. Mol. Genet. Metabolism 110, 98–101. 10.1016/j.ymgme.2013.07.006 23916421

[B5] Grünert S. C. Wendel U. Lindner M. Leichsenring M. Schwab K. O. Vockley J. (2012). Clinical and Neurocognitive Outcome in Symptomatic Isovaleric Acidemia. Orphanet J. Rare Dis. 7, 9. 10.1186/1750-1172-7-9 22277694 PMC3292949

[B6] Han L. S. Ye J. Qiu W. J. Gao X. L. Wang Y. Gu X. F. (2007). Selective Screening for Inborn Errors of Metabolism on Clinical Patients Using Tandem Mass Spectrometry in China: a Four-Year Report. J. Inherit. Metab. Dis. 30, 507–514. 10.1007/s10545-007-0543-9 17347912

[B7] Hasegawa Y. Iga M. Kimura M. Shigematsu Y. Yamaguchi S. (2005). Prenatal Diagnosis for Organic Acid Disorders Using Two Mass Spectrometric Methods, Gas Chromatography Mass Spectrometry and Tandem Mass Spectrometry. J. Chromatogr. B 823, 13–17. 10.1016/j.jchromb.2005.04.020 15908288

[B8] Hine D. G. Hack A. M. Goodman S. I. Tanaka K. (1986). Stable Isotope Dilution Analysis of Isovalerylglycine in Amniotic Fluid and Urine and its Application for the Prenatal Diagnosis of Isovaleric Acidemia. Pediatr. Res. 20, 222–226. 10.1203/00006450-198603000-00005 3703611

[B9] Hu Z. Yang J. Hu L. Zhao Y. Zhang C. Yang R. (2020). Screening and Clinical Analysis of Isovaleric Acidemia Newborn in Zhejiang Province. Zhejiang Da Xue Xue Bao Yi Xue Ban. 49, 556–564. 10.3785/j.issn.1008-9292.2020.10.02 33210480 PMC8800726

[B10] Ibarra-González I. Fernández-Lainez C. Guillén-López S. López-Mejía L. Belmont-Matínez L. Sokolsky T. D. (2020). Molecular Analysis Using Targeted Next Generation DNA Sequencing and Clinical Spectrum of Mexican Patients with Isovaleric Acidemia. Clin. Chim. Acta 501, 216–221. 10.1016/j.cca.2019.10.041 31707166

[B11] Ji X. Wang H. Ye J. Qiu W. Zhang H. Liang L. (2019). Prenatal Diagnosis of Methylmalonic Aciduria from Amniotic Fluid Using Genetic and Biochemical Approaches. Prenat. Diagn. 39, 993–997. 10.1002/pd.5519 31278756

[B12] Kasper D. C. Ratschmann R. Metz T. F. Mechtler T. P. Möslinger D. Konstantopoulou V. (2010). The National Austrian Newborn Screening Program - Eight Years Experience with Mass Spectrometry. Past, Present, and Future Goals. Wien Klin. Wochenschr 122, 607–613. 10.1007/s00508-010-1457-3 20938748

[B13] Kimmoun A. Abboud G. Strazeck J. Merten M. Guéant J.-L. Feillet F. (2008). Acute Decompensation of Isovaleric Acidemia Induced by Graves' Disease. Intensive Care Med. 34, 2315–2316. 10.1007/s00134-008-1192-7 18607566

[B14] Kleijer W. J. Van Der Kraan M. Huijmans J. G. M. Van Den Heuvel C. M. M. Jakobs C. (1995). Prenatal Diagnosis of Isovaleric Acidaemia by Enzyme and Metabolite Assay in the First and Second Trimesters. Prenat. Diagn. 15, 527–533. 10.1002/pd.1970150605 7659686

[B15] Kölker S. Valayannopoulos V. Burlina A. B. Sykut-Cegielska J. Wijburg F. A. Teles E. L. (2015). The Phenotypic Spectrum of Organic Acidurias and Urea Cycle Disorders. Part 2: the Evolving Clinical Phenotype. J. Inherit. Metab. Dis. 38, 1059–1074. 10.1007/s10545-015-9840-x 25875216

[B16] Kumps A. Vamos E. Mardens Y. Abramowicz M. Genin J. Duez P. (2004). Assessment of an Electron-Impact GC-MS Method for Organic Acids and glycine Conjugates in Amniotic Fluid. J. Inherit. Metab. Dis. 27, 567–579. 10.1023/b:boli.0000042981.52186.a9 15669672

[B17] Lee Y.-W. Lee D. H. Vockley J. Kim N.-D. Lee Y. K. Ki C.-S. (2007). Different Spectrum of Mutations of Isovaleryl-CoA Dehydrogenase (IVD) Gene in Korean Patients with Isovaleric Acidemia. Mol. Genet. Metabolism 92, 71–77. 10.1016/j.ymgme.2007.05.003 PMC4136440 17576084

[B18] Lin W.-D. Wang C.-H. Lee C.-C. Lai C.-C. Tsai Y. Tsai F.-J. (2007). Genetic Mutation Profile of Isovaleric Acidemia Patients in Taiwan. Mol. Genet. Metabolism 90, 134–139. 10.1016/j.ymgme.2006.08.011 17027310

[B19] Lin Y. Chen D. Peng W. Wang K. Lin W. Zhuang J. (2020). Newborn Screening for Isovaleric Acidemia in Quanzhou, China. Clin. Chim. Acta 509, 25–29. 10.1016/j.cca.2020.06.010 32505769

[B20] McHugh D. M. S. Cameron C. A. Abdenur J. E. Abdulrahman M. Adair O. Al Nuaimi S. A. (2011). Clinical Validation of Cutoff Target Ranges in Newborn Screening of Metabolic Disorders by Tandem Mass Spectrometry: a Worldwide Collaborative Project. Genet. Med. 13, 230–254. 10.1097/GIM.0b013e31820d5e67 21325949

[B21] Roberts E. Duckett D. P. Lang G. D. (1988). Maternal Cell Contamination in Chorionic Villus Samples Assessed by Direct Preparations and Three Different Culture Methods. Prenat. Diagn. 8, 635–640. 10.1002/pd.1970080902 3211852

[B22] Shigematsu Y. Kikawa Y. Sudo M. Kanaoka H. Fujioka M. Dan M. (1991). Prenatal Diagnosis of Isovaleric Acidemia by Fast Atom Bombardment and Tandem Mass Spectrometry. Clin. Chim. Acta 203, 369–374. 10.1016/0009-8981(91)90310-9 1777996

[B23] Szymańska E. Jezela-Stanek A. Bogdańska A. Rokicki D. Ehmke vel Emczyńska-Seliga E. Pajdowska M. (2020). Long Term Follow-Up of Polish Patients with Isovaleric Aciduria. Clinical and Molecular Delineation of Isovaleric Aciduria. Diagnostics 10, 738. 10.3390/diagnostics10100738 PMC7598207 32977617

[B24] Tanacan A. Gurbuz B. Aydin E. Erden M. Coskun T. Beksac M. (2019). Prenatal Diagnosis of Organic Acidemias at a Tertiary Center. Balk. J. Med. Genet. 22, 29–34. 10.2478/bjmg-2019-0003 PMC6714333 31523617

[B25] Tanaka K. Budd M. A. Efron M. L. Isselbacher K. J. (1966). Isovaleric Acidemia: a New Genetic Defect of Leucine Metabolism. Proc. Natl. Acad. Sci. U.S.A. 56, 236–242. 10.1073/pnas.56.1.236 5229850 PMC285701

[B26] Vockley J. Ensenauer R. (2006). Isovaleric Acidemia: New Aspects of Genetic and Phenotypic Heterogeneity. Am. J. Med. Genet. 142c, 95–103. 10.1002/ajmg.c.30089 16602101 PMC2652706

[B27] Xiao B. Qiu W. Ye J. Zhang H. Zhu H. Wang L. (2020). Prenatal Diagnosis of Glutaric Acidemia I Based on Amniotic Fluid Samples in 42 Families Using Genetic and Biochemical Approaches. Front. Genet. 11, 496. 10.3389/fgene.2020.00496 32508882 PMC7251148

