# Supplementary material for: Prenatal Diagnosis of Isovaleric Acidemia From Amniotic Fluid Using Genetic and Biochemical Approaches
Source: Front Genet. 2022 Jun 30;13:898860. doi: 10.3389/fgene.2022.898860 (PMC9280075; doi:10.3389/fgene.2022.898860)
Supplement: Supplementary file 1 [file DataSheet1.PDF]

Metabolite measurement using tandem mass spectrometry (MS/MS) and gas chromatography/mass spectrometry (GC/MS) in amniotic fluid samples is the same as that in the blood and urine, respectively. Only the reference range is different, thus, there is no other method developed.

Acylcarnitines analysis by MS/MS

3μl of amniotic fluid sample was placed on a 96-well filter plate. 100μl of methanol containing the isotopic internal standard was added to each well. After 20 min at room temperature, the fluid was centrifuged into a 96-well polypropylene plate. All wells were dried at 55°C under a nitrogen flow and to each 70μl of N-butanol hydrochloride was added. The plate was then covered with a Teflon membrane and placed in an incubator at 65°Cfor 15 min. After drying at 55°C under a nitrogen flow, 100μl of acetonitrile–water (4:1v/v) was added to each well and the plate was covered with aluminium membrane. 10μl of the final sample was injected into the MS/MS. The acylcarnitines were tested with the precursor scan mode and the levels of acylcarnitines were automatically calculated according to the assigned values of the internal standards using the software. The experimental parameters are listed in Supplementary Table 1.

Organic acids analysis by GC/MS

2 ml of amniotic fluid sample was added with 1ml ethanol and 20μl heptadecanoic acid. The mixture was then centrifuged at 4000 r/min for 5min. The supernatant was dried under a nitrogen stream at 60 °C. The dry residue was re-dissolved in a solution of 100μl of silylation reagent, followed by reaction at 80°C for 30 min. 1μl of the final derivatized aliquot was injected into the GC/MS. These organic acids were tested with selected ion monitoring mode and the concentrations of organic acids were calculated using GC-MS Solution v2.40 software. The experimental parameters are listed in Supplementary Table 2.

Supplementary Table 1 Parameters for MS/MS analysis (precursor scan mode, product ion mass-to-charge ratio：85.1)

Number	Parameter			Internal standard			Reference range	
	Analyte Name	Abbreviation	Parent ion mass-to-charge ratio（m/z）	Abbreviation	Mass-to-charge ratio（m/z）	Concentration	Low	High
1	Free carnitine	C0	218.2	d9-C0	227.2	0.76	10.00	60.00
2	Acetylcarnitine	C2	260.3	d3-C2	263.3	0.19	6.00	30.00
3	Propionylcarnitine	C3	274.3	d3-C3	277.3	0.04	0.50	4.00
4	Malonylcarnitine	C3DC	360.2	d3-C8	347.4	0.04	0.03	0.40
5	Butylcarnitine	C4	288.3	d3-C4	291.3	0.04	0.06	0.50
6	3-hydrobutylcarnitine	C4-OH	304.3	d9-C5	311.3	0.04	0.02	0.35
7	Methylmalonylcarnitine	C4DC	374.4	d3-C8	347.3	0.04	0.20	1.20
8	Isovalerylcarnitine	C5	302.3	d9-C5	311.3	0.04	0.04	1.00
9	Tiglylcarnitine	C5:1	300.3	d9-C5	311.3	0.04	0.00	0.10
10	3-hydroxyisovlerylcarnitine	C5-OH	318.3	d3-C5OH	321.3	0.04	0.06	0.60
11	Glutarylcarnitine	C5DC	388.4	d3-C5DC	391.4	0.08	0.00	0.20
12	Hexanoylcarnitine	C6	316.3	d9-C5	311.3	0.04	0.01	0.15
13	Hexenoylcarnitine	C6:1	314.3	d9-C5	311.3	0.04	0.01	0.10
14	Adipylcarnitine	C6DC	402.4	d9-C12	409.4	0.04	0.00	0.06
15	Octanoylcarnitine	C8	344.4	d3-C8	347.3	0.04	0.01	0.30

16	Octenoylcarnitine	C8:1	342.4	d3-C8	347.3	0.04	0.03	0.50
17	Suberylcarnitine	C8DC	430.4	d9-C14	437.5	0.04	0.00	0.04
18	Decanoylcarnitine	C10	372.4	d3-C8	347.3	0.04	0.02	0.50
19	Decenoylcarnitine	C10:1	370.4	d3-C8	348.3	0.04	0.03	0.45
20	Lauroylcarnitine(Dodecanoylcarnitine)	C12	400.4	d9-C12	409.4	0.04	0.02	0.20
21	Dodecenoylcarnitine	C12:1	398.3	d9-C12	409.4	0.04	0.02	0.20
22	Myristoylcarnitine	C14	428.5	d9-C14	437.5	0.04	0.02	0.25
23	myristoleylcarnitine	C14:1	426.5	d9-C14	438.5	0.04	0.01	0.30
24	3-hydromyristoylcarnitine	C14-OH	444.5	d9-C14	440.5	0.04	0.00	0.06
25	Palmitoylcarnitine	C16	456.5	d3-C16	459.5	0.08	0.30	2.00
26	Hexadecenoylcarnitine	C16:1	454.5	d3-C16	459.5	0.08	0.02	0.20
27	3-Hydroxy-hexadecanoylcarnitine	C16-OH	472.5	d3-C16	459.5	0.08	0.00	0.05
28	Octadacanoylcarnitine	C18	484.5	d3-C18	487.4	0.08	0.20	1.20
29	Oleylcarnitine	C18:1	482.5	d3-C18	487.4	0.08	0.30	1.80
30	3-Hydroxy-octadecanoylcarnitine	C18-OH	500.5	d3-C18	487.4	0.08	0.00	0.03
31	C3/C0	C3/C0					0.03	0.15
32	C3/C2	C3/C2					0.04	0.25
33	C4/C2	C4/C2					0.00	0.05

34	C5/C2	C5/C2	0.01	0.08
35	C5-OH/C2	C5-OH/C2	0.00	0.05
36	C5DC/C8	C5DC/C8	0.10	2.50
37	C8/C2	C8/C2	0.00	0.02
38	C14:1/C8:1	C14:1/C8:1	0.07	2.00
39	(C16+C18:1)/C2	(C16+C18:1)/C2	0.06	0.25
40	C0/(C16+C18)	C0/(C16+C18)	10.00	40.00

Supplementary Table 2 Parameters for GC/MS analysis (selected ion monitoring mode)

Number	Compound Name	MW	M.U	C.R. TIME	Q.ION	C.ION	Reference range	
							Low	High
1	Lactic-2	234	10.57	9.55	219	191	0.60	1140.00
2	2-OH-isobutyric-2	248	10.69	9.95	205	233	0.00	10.00
3	Hexanoic-1	188	10.71	10.02	173	132	0.00	0.80
4	Glycolic-2	220	10.72	10.05	205	177	0.00	7.50
5	Oxalic-2	234	11.31	12.03	219	190	0.00	10.00
6	2-OH-butyric-2	248	11.32	12.07	205	233	0.80	45.00
7	Glyoxylic-OX-2	233	11.25	11.83	218	233	0.00	8.00
8	3-OH-propionic-2	234	11.35	12.47	177	219	0.00	35.00
9	Pyruvic-OX-2	247	11.49	12.64	232	247	20.00	700.00
10	Valproic(VPA)-1	216	11.5	12.67	201	174	0.00	1.15
11	3-OH-butyric-2	248	11.63	13.11	191	233	0.25	115.00
12	3-OH-isobutyric-2	248	11.64	13.14	177	233	0.00	100.00
13	2-OH-isovaleric-2	262	11.71	13.38	219	247	0.00	15.00
14	2-Methyl-3-OH-butyric-1-2	262	12.02	14.42	247	218	0.00	0.00
15	Malonic-2	248	12.05	14.52	233	133	0.00	1.52

16	3-OH-isovaleric-2	262	12.14	14.83	247	205	0.00	1.50
17	2-Keto-isovaleric-OX-2	275	12.14	14.83	260	232	0.00	20.00
18	Methylmalonic-2	262	12.19	15	247	218	0.00	0.80
19	Ethylhydracrylic-2	262	12.32	15.45	247	177	0.00	3.50
20	Urea-2	204	12.37	15.62	189	171	0.00	80.00
21	4-OH-butyric-2	248	12.38	15.66	204	233	0.00	0.00
22	2-OH-isocaproic-2	276	12.42	15.79	261	233	0.00	0.50
23	3-OH-valeric-2	262	12.42	15.79	233	247	0.00	0.50
24	Acetoacetic	246	12.49	16.03	231	246	0.00	0.00
25	2-OH-3-methylvaleric-2	276	12.5	16.07	261	233	0.00	0.00
26	Benzoic-1	194	12.53	16.17	179	105	0.00	15.00
27	Acetoacetic-OX-2	261	12.61	16.45	246	261	0.00	6.00
28	Octanoic-1	216	12.65	16.58	201	117	0.00	0.00
29	2-Keto-3-methylvaleric-OX-2	289	12.76	16.96	274	200	0.15	12.00
30	2-Methyl-3-OH-valeric-2(1)	276	12.76	16.96	203	115	0.00	2.00
31	Glycerol-3	308	12.82	17.17	205	218	0.70	40.00
32	Phosphoric-3	314	12.83	17.2	299	314	3.00	210.00
33	2-Methyl-3-OH-valeric-2(2)	276	12.84	17.24	203	115	0.00	2.00
34	Ethylmalonic-2	276	12.86	17.3	261	217	0.00	0.70
35	2-Keto-isocaproic-OX-2	289	12.91	17.48	274	200	0.80	40.00

36	Acetylglycine-1	189	12.92	17.51	130	174	0.00	20.00
37	Phenylacetic-1	208	13.03	17.89	193	164	0.00	0.00
38	Maleic-2	260	13.1	18.13	245	170	0.00	0.00
39	Succinic-2	262	13.16	18.33	247	172	5.00	45.00
40	Methylsuccinic-2	276	13.28	18.75	261	217	0.00	0.00
41	Glyceric-3	322	13.39	19.12	292	307	0.80	4.50
42	Uracil-2	256	13.46	19.37	241	256	0.00	1.00
43	Fumaric-2	260	13.48	19.43	143	245	2.00	18.00
44	Propionylglycine-1	203	13.59	19.81	188	159	0.00	0.00
45	Acetylglycine-1	261	13.72	20.26	218	246	0.00	0.00
46	Mevalonolactone-2	274	13.88	20.81	187	229	0.00	0.00
47	Mevalonolactone-1	202	13.93	20.98	187	157	0.00	0.00
48	Isobutyrylglycine-1	217	13.92	20.95	202	173	0.00	0.00
49	2-Propyl-3-OH-pentanoic (VPA)-2	304	13.93	20.98	275	289	0.00	0.00
50	Mesaconic (Methylfumaric)-2	274	14	21.22	259	184	0.00	0.00
51	Glutaric-2	276	14.04	21.35	261	158	0.00	2.50
52	3-Methylglutaconic-2	288	14.17	21.75	273	183	0.00	0.15
53	3-Methylglutaric-2	290	14.26	22.04	275	247	0.00	0.00
54	2-Propyl-3-ketopentanoic (VPA)-2	302	14.26	22.04	287	155	0.00	0.00
55	Propionylglycine-2	275	14.28	22.1	260	232	0.00	0.00

56	Isobutyrylglycine-2	289	14.3	22.16	274	100	0.00	0.00
57	2-Deoxytetronic	336	14.39	22.45	233	321	0.00	0.60
58	Butyrylglycine-1	217	14.42	22.54	202	173	0.00	0.00
59	3-Methylglutaconic-2	288	14.44	22.6	273	183	0.00	0.15
60	Glutaconic-2	274	14.48	22.73	259	230	0.00	0.30
61	Succinylacetone-OX-2(1)	227	14.53	22.89	212	227	0.00	0.00
62	Decanoic-1	244	14.6	23.11	229	145	0.00	0.00
63	2-Propyl-5-OH-pentanoic (VPA)-2	304	14.84	23.86	289	185	0.00	0.00
64	3-Methylglutaconic-2	288	14.84	23.86	273	183	0.00	1.20
65	Isovalerylglycine-1	231	14.88	23.99	216	189	0.00	0.00
66	Butyrylglycine-2	289	14.92	24.11	274	289	0.00	0.00
67	Malic-3	350	14.99	24.33	335	233	1.40	10.00
68	Adipic-2	290	15.1	24.68	275	111	0.00	0.40
69	Isovalerylglycine-2	303	15.2	24.99	288	176	0.00	0.00
70	2-Hexenedioic-2	288	15.22	25.06	273	288	0.00	1.20
71	5-Oxoproline-2(pyroglutamic)	273	15.34	25.43	258	230	0.00	35.00
72	3-methyladipic	304	15.39	25.59	289	204	0.00	0.00
73	Thiodiglycolic-2	294	15.41	25.65	204	294	0.00	0.00
74	2-Propyl-hydroxyglutaric(VPA)-2	318	15.51	25.97	303	259	0.00	0.00
75	7-OH-octanoic-2	304	15.51	25.97	289	260	0.00	0.00

76	5-OH-methyl-2-furoic-1	286	15.54	26.06	271	197	0.00	0.60
77	Tiglylglycine-2	301	15.64	26.38	286	301	0.00	0.00
78	3-Methylcrotonylglycine-1	229	15.64	26.38	214	229	0.00	0.30
79	Tiglylglycine-1	229	15.71	26.6	229	214	0.00	0.50
80	3-Methylcrotonylglycine-2	301	15.78	26.82	286	184	0.00	0.00
81	2-OH-glutaric-3	364	15.81	26.91	349	203	0.00	3.00
82	3-OH-glutaric-3	364	15.82	26.94	247	349	2.00	15.00
83	Phenyllactic-2	310	16	27.51	194	267	0.50	4.00
84	Pimelic-2	304	16.04	27.62	155	289	0.00	2.00
85	3-OH-3-methylglutaric-3	378	16.11	27.82	247	273	0.00	2.50
86	3-OH-phenylacetic-2	296	16.16	27.96	296	281	0.00	22.00
87	2-Ketoglutaric-OX-2(1)	377	16.35	28.5	362	377	4.00	60.00
88	4-OH-benzoic-2	282	16.37	28.56	267	282	0.00	100.00
89	4-OH-phenylacetic	296	16.48	28.87	296	281	0.00	10.00
90	2-Ketoglutaric-OX-2(2)	377	16.55	29.07	362	377	0.00	30.00
91	Hexanoylglycine-1	317	16.55	29.07	302	200	0.00	0.20
92	Phenylpyruvic-OX-2	323	16.62	29.27	308	147	0.00	0.00
93	N-Acetylaspartic-2	319	16.71	29.52	202	304	0.00	1.20
94	2-OH-adipic-3	378	16.79	29.75	261	363	0.00	5.00
95	Octenedioic-2	316	16.82	29.83	185	301	0.00	0.00

96	3-OH-adipic-3	378	16.91	30.09	363	247	0.00	0.00
97	Suberic-2	318	17.02	30.4	303	187	0.00	0.60
98	3-Methylglutaconic-2	360	17.14	30.74	345	360	0.00	0.00
99	2-Keto-adipic-OX-3	391	17.17	30.83	302	258	0.00	1.65
100	Aconitic-3	390	17.54	31.88	229	375	0.00	35.00
101	Orotic-3	372	17.58	31.99	357	254	0.00	1.00
102	Vanillic-2	312	17.72	32.39	312	297	0.00	1.00
103	Homovanillic-2(HVA)	326	17.83	32.7	326	311	0.00	1.50
104	Azelaic-2	332	18	33.18	317	152	0.00	4.00
105	Hippuric-2	323	18.2	33.69	323	308	0.00	0.50
106	Isocitric-4	480	18.36	34.11	245	465	7.00	30.00
107	Citric-4	480	18.37	34.13	273	465	350.00	1500.00
108	Homogentisic-3	384	18.5	34.47	384	341	0.00	0.00
109	Hippuric-1	251	18.51	34.49	236	206	0.00	13.00
110	Methylcitric-4(1)	494	18.62	34.77	389	479	0.00	0.50
111	3-(3-OH-phenyl)-3-OH-propionic-3	398	18.64	34.82	280	398	0.00	1.50
112	Methylcitric-4(2)	494	18.71	35	389	479	0.00	0.50
113	3-OH-octenedioic-3	404	18.79	35.21	233	389	0.00	6.00
114	3-OH-suberic-3	406	18.81	35.26	169	391	0.00	140.00
115	Vanilmandelic-3(VMA)	414	18.96	35.65	297	371	0.00	3.00

116	Sebacic-2	346	18.99	35.72	331	215	0.00	8.00
117	Decadienedionic-2	342	19.03	35.83	195	327	0.00	0.25
118	4-OH-phenyllactic(PHPLA)-2	398	19.19	36.24	308	293	4.00	25.00
119	4-OH-phenylpyruvic (PHPPA)-OX-2	411	19.51	37.06	396	179	0.00	2.00
120	2-OH-hippuric-3	411	19.73	37.63	396	411	0.00	2.00
121	Indole-3-acetic-2	319	19.84	37.91	202	319	0.00	10.00
122	Suberylglycine-2	375	20.26	38.93	360	229	0.00	0.50
123	Palmitic-1	328	20.46	39.4	313	269	10.00	250.00
124	2-OH-sebacic-3	419	20.59	39.7	317	391	0.00	0.52
125	3-OH-sebacic-3	434	20.71	39.98	303	233	0.00	0.00
126	2-OH-hippuric-2	339	20.86	40.34	324	206	0.00	0.15
127	Dodecanedioic-2	374	20.89	40.41	359	243	0.00	1.20
128	N-Acetyltyrosine-3	439	21.19	41.11	308	424	0.00	0.10
129	Uric-4	456	21.27	41.3	456	441	0.00	55.00
130	3,6-Epoxydodecanedioic-2	385	21.71	42.33	201	174	0.00	20.00
131	3-OH-dodecanedioic-3	462	22.62	44.34	233	447	0.00	0.00
132	3,6-Epoxytetradecanedioic-2	416	23.61	46.47	201	159	0.00	0.30

Abbreviation：Q- ion, selected ion for quantification; C-ion selected ion for confirmation
